# ABO antigen and secretor statuses are not associated with gut microbiota composition in 1,500 twins

**DOI:** 10.1186/s12864-016-3290-1

**Published:** 2016-11-21

**Authors:** Emily R. Davenport, Julia K. Goodrich, Jordana T. Bell, Tim D. Spector, Ruth E. Ley, Andrew G. Clark

**Affiliations:** 1 Department of Molecular Biology & Genetics, Cornell University, Ithaca, NY USA; 2 Department of Twin Research and Genetic Epidemiology, King’s College London, London, UK; 3 Department of Microbiome Science, Max Planck Institute for Developmental Biology, Tübigen, Germany

**Keywords:** Microbiome, Heritability, Blood group antigens, Secretor status, ABO, FUT2

## Abstract

**Background:**

Host genetics is one of several factors known to shape human gut microbiome composition, however, the physiological processes underlying the heritability are largely unknown. Inter-individual differences in host factors secreted into the gut lumen may lead to variation in microbiome composition. One such factor is the ABO antigen. This molecule is not only expressed on the surface of red blood cells, but is also secreted from mucosal surfaces in individuals containing an intact *FUT2* gene (secretors). Previous studies report differences in microbiome composition across ABO and secretor genotypes. However, due to methodological limitations, the specific bacterial taxa involved remain unknown.

**Results:**

Here, we sought to determine the relationship of the microbiota to ABO blood group and secretor status in a large panel of 1503 individuals from a cohort of twins from the United Kingdom. Contrary to previous reports, robust associations between either ABO or secretor phenotypes and gut microbiome composition were not detected. Overall community structure, diversity, and the relative abundances of individual taxa were not significantly associated with ABO or secretor status. Additionally, joint-modeling approaches were unsuccessful in identifying combinations of taxa that were predictive of ABO or secretor status.

**Conclusions:**

Despite previous reports, the taxonomic composition of the microbiota does not appear to be strongly associated with ABO or secretor status in 1503 individuals from the United Kingdom. These results highlight the importance of replicating microbiome-associated traits in large, well-powered cohorts to ensure results are robust.

**Electronic supplementary material:**

The online version of this article (doi:10.1186/s12864-016-3290-1) contains supplementary material, which is available to authorized users.

## Background

Host genetics shapes the composition of the gut microbiome [1–4]. The mechanisms by which this occurs are not completely understood, but could include immune regulation, host digestive enzyme production, and cell surface antigen presentation. One such antigen that may play a role is encoded by *ABO* and is classically known as the major red blood cell histocompatibility molecule for blood transfusions. ABO and other blood group antigens are not only found on the surface of red blood cells, but are also secreted from mucosal tissues in individuals with an intact *FUT2* gene (secretors). In secretors, blood group antigens expressed in the gut interact with certain members of the microbiota. For example, *H. pylori* tethers itself to the mucosal lining using blood group antigens [5] and bacteria from stool express enzymes that degrade ABO to provide an energy source [6–8]. Therefore, host genetic variation in both *ABO* and *FUT2* may have broad effects on microbiome composition.

Additionally, *ABO* and *FUT2* variants are risk factors for a number of different diseases, including Crohn’s disease [9, 10], AIDs [11], Type 1 diabetes [12], and infectious diseases [13–15]. The etiology underlying many of these associations is unknown, but evidence points towards a role of the gut microbiome. For example, the intestinal microbiome was found to vary by both Crohn’s disease status and *FUT2* genotype [16]. Furthermore, metagenomes from individuals discordant for *FUT2* genotype revealed differences in gene content related to energy metabolism [17]. Disease status for both AIDS [18–20] and Type 1 diabetes [21–23] are associated with gut microbial composition differences between cases and controls. Finally, microbiome composition affects susceptibility and disease progression for infectious diseases, including norovirus infection [24], influenza [25], and cholera [26] – all diseases for which ABO or secretor status are risk factors. Therefore, an open question is whether host genetic variation in *ABO* and *FUT2* mediates disease risk through the gut microbiome and which taxa are key players in this process.

Results from two recent studies lend support to this hypothesis. Microbiome composition differed according to secretor status in a cohort of 71 individuals from Finland [27]. The microbiomes of non-secretors were more diverse overall; however, non-secretors contained significantly more species of bifidobacteria than secretors. In a follow up study examining ABO status only in secretors, B and AB individuals clustered separately from A and O individuals in ordination analysis of total microbiome composition [28]. While these studies provide proof of principle that genetic variation in *ABO* and *FUT2* can be associated with microbiome composition, the methods employed in these studies have limited resolution. It is unclear which bacteria drive the observed patterns and whether those are the same taxa that are associated with risk for diseases linked to *ABO* or *FUT2* variation.

Here, we sought to determine if ABO antigen and secretor phenotypes were associated with gut microbiome composition in a panel of 1503 individuals as part of the United Kingdom adult Twin Health Registry (TwinsUK) cohort, where comprehensive microbiome, disease, and genotype data are available [1, 29, 30]. We examined broad community composition using ordination techniques and diversity measurements, presence/absence or relative abundance of individual bacterial taxa using linear mixed models, and we applied classification techniques to jointly model taxa in relation to ABO or secretor status. Contrary to previous findings, we do not observe robust associations of the gut microbiome to ABO or secretor status.

## Results

### Community composition not significantly associated to ABO or secretor status

**Fig. 1:**
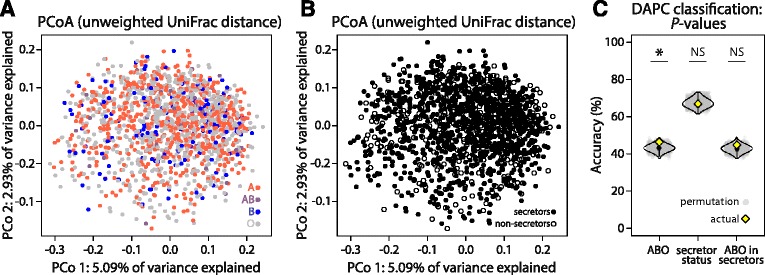
Neither ABO or secretor status associated with broad compositional differences of the gut microbiota in the TwinsUK. None of the top 100 principal coordinates (PCs) from principal coordinate analysis of unweighted UniFrac distance are significantly associated with either ABO or secretor status. The first two PCs are shown, colored by ABO status (**a**) and secretor status (**b**). **c** Discriminant analysis of PCA (DAPC) is largely unsuccessful at predicting ABO or secretor status from microbiome data. The mean accuracy from 5-fold cross validation is plotted for ABO status, secretor status, and ABO status only in secreting individuals (yellow). Significance was determined by comparing the accuracy of each test to the accuracies of permuted data, which took into account twin relationships (gray). Significance codes: *P* ≤ 0.05 = *, not significant = NS

We first sought to determine whether there were broad compositional differences in the microbiome associated either with ABO status, secretor status, or ABO status in secreting individuals only (Table 1, Additional file 1: Table S1). In a recent study, individuals with B alleles clustered separately from A and O individuals in redundancy analysis of microbiome composition, as determined by denaturing gradient gel electrophoresis (DGGE) [28]. We applied several ordination approaches, but were unable to recapitulate those findings. First, we ran principal coordinate analysis using three beta-diversity metrics: unweighted UniFrac distance (Fig. 1), weighted UniFrac distance, and Bray-Curtis dissimilarity (Additional file 2: Figure S1). None of the top 100 principal coordinates (PCs) from any diversity metric tested were significantly associated with either ABO status or secretor status (linear model, *q* > 0.05, Additional file 3: Table S2).

**Table 1:** ABO and secretor phenotypes in the TwinsUK dataset

	Total	Secretors		Non-secretors		Unknown	
		Female	Male	Female	Male	Female	Male
A	606	405	35	137	14	12	3
AB	40	20	6	9	3	2	0
B	140	96	7	30	3	2	2
O	717	449	41	175	22	27	3
Total	1503	970	89	351	42	43	8

Additionally, we applied principal components analysis (PCA) to both i) covariate-corrected, transformed relative abundance data for 835 operational taxonomic units (OTUs) present in at least 50% of individuals and ii) presence/absence data for all OTUs. Again, none of the top 100 principal components for each analysis were significantly associated with either ABO status or secretor status (linear model *q* > 0.05, Additional file 3: Table S2, Additional file 4: Figure S2).

Finally, we applied discriminant analysis of principal components (DAPC) to microbiome data [31]. This method was originally designed for analyzing structure in large genotype datasets, where PCA reduces data dimensionality and then discriminant analysis identifies the components that have high inter-group variability. Here, we use it to determine if microbiome composition can reliably distinguish individuals based on of their secretor or ABO status (in all individuals and secretors only). We ran DAPC using a 5-fold cross validation scheme to determine the accuracy of group classification. To assess significance, 1000 permutations of ABO or secretor status across individuals were run to generate a distribution of accuracies expected by chance, while controlling for twin relationships in the data (see methods). DAPC was not successful at predicting either secretor status (median accuracy = 67%, *P* > 0.05) or ABO status in secretors (median accuracy = 45%, *P* > 0.05, Fig. 1). Prediction for ABO status in all individuals was marginally significant when compared to permuted data (*P* = 0.035); however, accuracy was low (median accuracy = 46%). Through PCoA, PCA, and DAPC we fail to find evidence that there are broad compositional differences between individuals based on ABO status, secretor status, or ABO status in only secreting individuals.

### Microbiome diversity not associated with ABO or secretor status

**Fig. 2:**
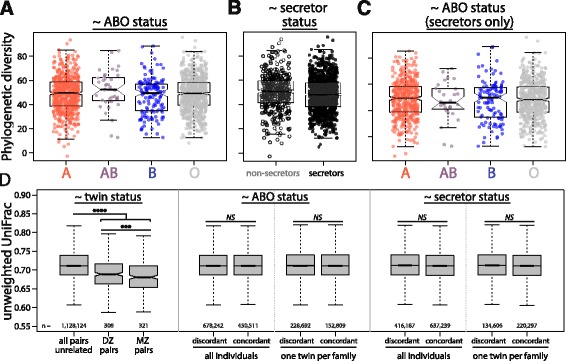
Microbiome diversity does not significantly differ by ABO or secretor status. Within sample diversity (Faith’s phylogenic diversity) is not significantly different (*P* > 0.05) across the ABO groups in all individuals (**a**), secretors versus non-secretors (**b**), or across ABO groups in only secreting individuals (**c**). **d** Microbiomes are more similar for siblings versus pairs of unrelated individuals, as measured by unweighted UniFrac distance. Additionally, pairs of monozygotic twins have significantly more similar microbiomes than dizygotic twins. However, microbiomes of pairs of individuals concordant for either ABO or secretor status are not more similar than for pairs of individuals who are discordant. This holds true when all individuals in the dataset are considered (“all individuals”) or when only one individual from each twin pair is examined (“one twin per family”). The total number of pairs of individual within each boxplot is indicated with “*n* = “. Significance codes: *P* ≤ 0.05 = *, *P* ≤ 0.01 = **, *P* ≤ 0.001 = ***, *P* ≤ 0.0001 = ****, not significant = NS

While ABO or secretor status may not determine overall microbiome composition consistently across individuals, they may be associated with differences in microbial diversity. A previous study reported evidence of this, where non-secreting individuals had higher species richness than secreting individuals [27]. In the TwinsUK dataset, linear mixed models were employed to model alpha diversity as a function of ABO status, secretor status, or ABO status in secreting individuals only (see methods) considering five different diversity metrics: Faith’s phylogenic diversity, number of observed OTUs, the Chao1 richness estimator, the Gini index, and the Shannon diversity index. No alpha diversity metric significantly differed by ABO status, secretor status, or by ABO status only in secretor, even before applying a correction for multiple testing (linear mixed model, *P* > 0.05, Fig. 2, Additional file 5: Figure S3).

When the effect of secretor status on diversity is examined within each ABO class individually, non-secreting AB individuals generally have higher diversity and evenness than secreting individuals, but non-secreting B individuals have lower diversity and evenness than secretors (linear mixed model, *P* ≤ 0.05, Additional file 6: Figure S4). All of the significant within class comparisons involve expression of at least one B allele; however, the direction of effect differs between secretors and non-secretors across comparisons. It is also important to note that the AB and B classes represent the two smallest ABO blood groups in the dataset, and therefore may be more susceptible to noise (*n*_*AB*_ = 38, *n*_*B*_ = 136 vs. *n*_*O*_ = 687, *n*_*A*_ = 591). Therefore, while there may be differences in diversity of the gut microbiome by secretor status in individuals that express B alleles, we do not find evidence to suggest that diversity differs consistently either due to secretor status or by an individual’s ABO antigen expression.

If ABO or secretor status influences the composition of the microbiome, we might expect that individuals concordant for either status would have more similar microbiomes than discordant individuals. To determine if this was the case, we compared average pairwise beta-diversity stratified by concordance for ABO or secretor status using three different diversity metrics. We considered two beta-diversity metrics that incorporate bacterial phylogenic information (weighted and unweighted UniFrac distance [32]) and one that does not (Bray Curtis dissimilarity [33]). A previous study using twins from the same cohort demonstrated that pairs of twins have more similar microbiomes than pairs of unrelated individuals on average [1]. Additionally, monozygotic twins have more similar microbiomes than dizygotic twins, pointing to a role for host genetics in determining gut microbiome composition. First, we recapitulate these results in this expanded dataset, which includes 629 pairs of twins. Pairs of twins have significantly lower beta-diversity than pairs of unrelated individuals for all beta-diversity metrics considered (permutation *P* < 0.0001, Fig. 2, Additional file 7: Figure S5). Additionally, pairs of monozygotic (MZ) twins have significantly lower beta-diversity than pairs of dizygotic (DZ) twins (permutation *P* < 0.001 unweighted UniFrac distance, *P* < 0.01 weighted UniFrac distance, *P* < 0.05 Bray Curtis dissimilarity).

Next, we stratified beta-diversity by concordance for either ABO or secretor status. No significant differences exist for any metric examined (permutation *P* > 0.05, Fig. 2, Additional file 7: Figure S5). First, we categorized all pairs of individuals as either concordant or discordant for ABO or secretor status, including all twin pairs. However, twins on average have more similar microbiomes due to shared environment and genetics, and are more likely to have concordant *ABO* or *FUT2* genotypes. To ensure that including twin pairs did not introduce bias, one twin from each twin pair was eliminated and the analysis was repeated. Again, no significant differences in beta-diversity were evident between individuals either concordant or discordant for ABO or secretor status. Therefore, not only do we fail to find evidence that overall microbiome diversity differs by ABO or secretor status, but we also do not find evidence that individuals who share the same genotypes at these loci have more similar microbiomes than individuals who do not.

### Relative abundance or presence/absence of individual bacterial taxa not associated with ABO or secretor status

The above analyses aimed to identify fairly broad compositional changes in the microbiome. While these are not apparent in our dataset, relative abundances of individual bacterial taxa may differ according to ABO or secretor status. Bifidobacteria species in particular have been observed to differ in individuals according to their secretor status [27] and also in breast-fed infants according to their mother’s secretor status [34]. We set out to test whether we could identify whether bifidobacteria or other bacterial taxa differ in relative abundance or presence/absence according to ABO or secretor status.

To identify common taxa that are differentially abundant according to ABO or secretor status, we implemented linear mixed models that include terms to account for the relatedness of twin pairs (see methods). We considered nine different models in total. We started with models that included terms for either ABO status or secretor status. Additionally, we considered models where individuals possessing B alleles were contrasted to other ABO groups, as previous studies observed broad microbial composition differences between B individuals and others [28]. Finally, we also tested for association using models that only consider ABO status in secretors or that include an interaction term between ABO and secretor status.

**Fig. 3:**
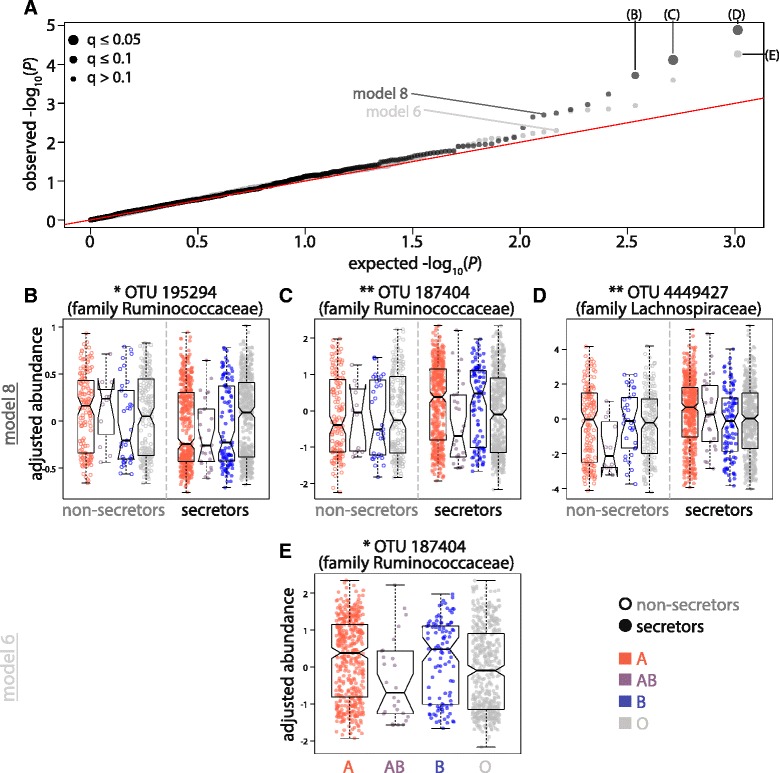
The relative abundances of several Firmicutes OTUs differ in secreting individuals. **a** QQ-plot displaying the expected –log_10_(*P*-value) compared to the –log_10_(*P*-value) for all taxa tested in linear mixed models 6 (light gray points) and 8 (dark gray points). Model 6 identified taxa differentially abundant across the four ABO classes in secreting individuals only while model 8 incorporated interaction terms between ABO groups and secretor status. All differentially abundant taxa passing a significance threshold of *q* ≤ 0.1 are indicated with larger point sizes and the distributions of covariate-corrected, transformed relative abundances of those taxa are displayed in (**b**-**e**)

Three OTUs were differentially abundant across ABO groups, according to two of the nine models tested at a moderate q-value threshold (linear mixed model *q* ≤ 0.1, Fig. 3, Additional file 8: Table S3, Additional file 9: Figure S6). OTU 187404, which is from the family Ruminococcaceae, has higher relative abundance in A and B secreting individuals than AB or O secretors (model 6). This same OTU is also differentially abundant in the model considering an interaction effect between ABO and secretor status (model 8). Two additional OTUs were differentially abundant according to model 8, including another OTU from the family Ruminococcaceae (*q* ≤ 0.1) and one from the family Lachnospiraceae (*q* ≤ 0.05). In the models above, q-values were calculated across all taxa tested within each model separately. If all comparisons are considered, no q-value surpasses a lenient *q* ≤ 0.1 threshold. Therefore, there is not strong evidence to support the hypothesis that relative abundances of common bacterial taxa are associated with either ABO or secretor status.

Additionally, we modified this analysis in order to control for ancestry by including the top five principal components (PCs) computed using genome-wide single nucleotide polymorphism (SNP) data as covariates in each of the nine models (see methods, Additional file 10: Figure S7, Additional file 11: Table S4). Results were largely robust with respect to the inclusion of PCs, with high correlation of P-values between analyses for each of the nine models (*r* ^*2*^ for models 1-9: 0.95, 0.93, 0.94, 0.93, 0.96, 0.97, 0.98, 0.94, 0.98). This was to be expected, as self-reported ancestry for a subset of the cohort revealed a largely homogenous population (*n* = 890: “White” = 97%, “Black” = 0.7%, “Mixed” = 0.7%, “Asian” = 0.3%, and “Other” = 0.3%). Therefore, differences in ancestry within the population are not masking more subtle genetic effects.

In addition to testing relative abundance differences across ABO or secretor groups, we also tested whether certain OTUs were more commonly observed in some ABO or secretor groups than others by examining presence/absence across individuals. To do so, Chi-squared tests of independence were applied to any OTU that was present in at least 10% of individuals (1692 OTUs), considering ABO status, secretor status, or ABO status in only secreting individuals. Presence/absence was not significantly different across ABO status groups for any of the 1692 OTUs tested at a lenient q-value threshold (*q* < 0.1, Additional file 12: Table S5). The presence and absence of two OTUs varied significantly according to secretor status (permutation *P* < 0.001), including OTU 195548 from order Clostridiales and OTU 365385 from genus *Bifidobacterium*, which were both more common in non-secretors than secretors. Additionally, presence of OTUs 4443846 (family Lachnospiraceae) and 592616 (family Erysipelotrichaceae) varied by ABO status in secreting individuals. However, relative abundances of these four OTUs are very low in the dataset (maximum relative abundance across all individuals varies from 0.04 - 5%), calling into question whether these are true biological results or if they are spurious results due to low sampling of rare species during sequencing.

In previous studies, *Bifidobacterium* species varied according to secretor status. Abundance varied in stool not only in accordance with an individual’s secretor status [27], but also in breastfed infants, when stratified by their mother’s secretor status [34]. We did not observe significant associations of any OTUs belonging to the genus *Bifidobacterium* or the collapsed genus *Bifidobacterium* (Additional file 8: Table S3); however, recent studies suggest a potential confounder may exist. *Bifidobacterium* relative abundance has been associated with variants near the lactase gene (*LCT*) [2, 35], which encodes for an enzyme that breaks down lactose to galactose and glucose in the small intestine. Derived mutations in enhancer regions near the lactase gene confer a lactase persistence phenotype, where individuals are able to degrade lactose into adulthood [36, 37]. The mechanism underlying the association between host variation near *LCT* and *Bifidobacterium* relative abundance is not understood, however, this association is evident in the TwinsUK cohort [35]. Therefore, it may be important to account for this confounder in analysis in order to identify differences in bifidobacterial content across ABO or secretor status groups. Unlike many other studies involving microbiome cohorts, the individuals in this study have genome-wide variant data available and we can take this potential confounder into account.

To take variation near *LCT* into account, the relative abundances of bifidobacterial taxa were modeled including a fixed effect term for rs1446585, a SNP near *LCT* that was associated with bifidobacterial relative abundance in a larger cohort of the UK twins [35]. Linear mixed models were used to examine whether bifidobacterial relative abundance varied by ABO status, secretor status, or ABO status in secretors while taking into account host genetic variation at this locus. Significant associations of bacterial relative abundance with ABO or secretor status were not detected for any of the common taxa examined, which included 6 OTUs belonging to genus *Bifidobacterium* and the collapsed taxonomic relative abundances for genus *Bifidobacterium*, family Bifidobacteriaceae, and order Bifidobacteriales (adjusted *P* > 0.05, Additional file 13: Table S6). Therefore, we find no evidence that bifidobacterial relative abundance differs according to ABO or secretor status in our dataset, even after taking into account the potential confounding effect of host genetic variation in *LCT*.

### Joint modeling approaches fail to identify bacteria predictive of ABO or secretor status

Large-scale microbiome composition, diversity, and relative abundance differences between ABO or secretor classes are not apparent in our dataset. However, there may be subtle differences across multiple bacterial taxa that are predictive of ABO or secretor status. These differences may be dwarfed by broader compositional patterns in PCoA and their effect sizes may be too small to detect via linear models with our sample size. However, machine-learning techniques may be able to identify these predictive taxa in combination. Therefore, we ran two different machine-learning techniques to identify bacterial taxa predictive of ABO or secretor status.

First, we applied random forests to the microbiome data. The random forest approach identifies collections of variables (taxa) that accurately predict classes of a dependent variable (ABO status, secretor status, or ABO status only in secretors). The random forests algorithm was benchmarked as achieving the best performance of several classifiers when applied to microbiome data [38], and it has been used successfully to identify gut taxa predictive age [39], individuals within a family [40], and future Crohn’s disease severity [41]. Here, we applied random forests to all the relative abundances of OTUs present in at least 50% of individuals using two strategies. First, trees were built using data from all individuals. For all models, the out-of-bag (OOB) error rates were high (OOB error: ABO status = 52–55%, secretor status = 27%, Additional file 14: Figure S8), and individuals classified consistently to the most common classes in the dataset (A or O for ABO status, and as secretors for secretor status). Uneven group sizes can lead to random forests favoring the majority classes [42], and this is potentially an issue in our dataset, where there are many more A and O individuals than AB or B (606 A and 717 O vs. 140 B and 40 AB individuals) and many more secretors than non-secretors (1059 S vs. 393 NS).

To combat this issue we employed a second strategy. Group sizes were down-sampled during random forests to the smallest group size, so that each class was equally represented during model building. Although classification was less skewed towards the common groups using this procedure, the error rates remained high (OOB error: ABO status = 62–66%, secretor status = 29%, Additional file 14: Figure S8). Therefore, the random forests method was unable to identify sets of bacterial taxa that in combination could predict ABO or secretor status in our dataset.

Our random forests models only included the relative abundances of OTUs present in at least half of the individuals in our dataset. However, methodology has recently been developed for multi-group classification of sparse data, of which gut microbiome data is a prime example. Multi-group sparse discriminant analysis (MGSDA) estimates canonical vectors directly from sparse data in the case where there are many more variables than subjects [43]. Here we use it to identify taxa in the gut microbiome predictive of ABO status, secretor status, or ABO status in secreting individuals only. We applied the algorithm to two different bacterial datasets for each ABO/secretor status comparison. First, we input all common bacterial OTUs and collapsed taxa to build the model. In addition, we built models that included all OTUs that were present in at least 10% of individuals in the study. In all cases, the accuracy of classification was poor, similar to the accuracies observed for the random forests method (accuracy ABO status = 45–47%, secretor status = 73%, ABO in secretors = 43–49%, Additional file 15: Table S7). MGSDA identified features predictive of ABO status for certain models (Additional file 16: Table S8). For example, the same three OTUs showing differential relative abundance according to ABO and secretor phenotypes through linear mixed model 8 were predictive of ABO status in secreting individuals through MGSDA (OTUs 187404, 4449427, and 195294). That being said, the low overall classification accuracy of these models indicates that these bacteria are not strongly predictive of ABO or secretor status, either individually or when considered in combination.

### Results recapitulated in “healthy” individuals

ABO antigen and secretor status are known risk factors for a number of diseases, many of which have also been associated with microbiome composition in the gut. Therefore, the inclusion of both healthy and potentially diseased individuals may pose a problem, as purely genetic associations between the microbiome and ABO or secretor status may be masked by larger microbiome shifts due to disease. Full medical histories are unavailable for our subjects; therefore we used body mass index (BMI) as a proxy for health, as obesity has comorbidities with a number of other diseases. We repeated the following analyses using a “healthy” subset of our cohort (consisting of individuals whose BMIs were less than 25 (*n* = 679)), and found our results from the full dataset were recapitulated: principal coordinates analysis, discriminant analysis of PCA, alpha diversity characterizations, beta-diversity characterizations, and linear mixed models of common taxa (Additional file 17: Figure S9, Additional file 18: Table S9).

## Discussion

In this study, we do not find evidence that either ABO antigen or secretor phenotypes are associated with overall fecal microbial community composition, diversity, or the relative abundances of individual bacterial taxa in a large panel of 1503 individuals from the UK. We aimed to be exhaustive in our methodology, ensuring that our results were not driven by choice of one particular statistical approach or metric. To that end, we examined multiple different ordination methods, diversity metrics, tests of differential relative abundance, and machine learning algorithms. Additionally, one strength of the TwinsUK cohort is the availability of genome-wide genotype data, which allowed us to adjust for genetic confounders that might obscure our ability to detect associations. Even when lenient multiple testing thresholds are applied, we are unable to replicate previous findings linking host genetic variation in *ABO* and *FUT2* to gut microbial composition [27, 28].

While associations between *ABO* and *FUT2* with the microbiome were not apparent in our dataset, there are a number of caveats that are important to consider. First, although our sample size is more than 20 times larger than previous studies [27, 28], we cannot exclude minor effects and may have insufficient power to detect taxa that show only slight differential relative abundances between groups. Additionally, when examining the relative abundances of individual bacteria, we eliminated any taxa present in fewer than 10% of individuals in our cohort. It is possible that very rare taxa may be able to distinguish ABO or secretor status, particularly if they are more abundant in classes for which we had smaller sample size (AB or B individuals and non-secretors). Whether taxa with small relative abundance differences between classes or that are very rare have broader biological implications remains an open question.

Additionally, environmental context may prove to be important in determining whether an individual’s ABO or secretor status influences microbiome composition. The individuals included in our study consist of mostly adult, female twins living in UK (average age = 61 years, 91% female). The gut microbiome changes with age [44, 45], and the average age of our dataset is older than the previous studies (average age: 61 vs. 45 years) [27, 28]. Differences in physiology, diet, or activity level between age groups may lead to microbiome composition differences that swamp out signals of association with *ABO* or *FUT2*.

Another environmental variable that may be important to consider is diet. Inter-individual differences in diet profoundly influence gut microbiome composition [46]. Individuals in our study provided samples without any dietary restriction or guidelines, and diet likely varied widely across the cohort. Notably, a strong diet by genotype interaction has been observed in humanized mouse models examining the effect of secretor status on gut microbial composition [47]. In this model, both microbial evenness and the relative abundances of several taxa differed between secreting and non-secreting mice on a standard chow diet. However, those differences were eliminated when mice were switched to a diet low in complex polysaccharides but high in simple glucose. Therefore, dietary variation in our study potentially masks the effect secretor status may play in determining gut microbiome composition.

Finally, the effects of ABO or secretor status may be more prominent in a disease context, where host physiology and overall microbiome composition is altered. For example, a genotype by disease interaction was observed for the association of secretor status and Crohn’s disease to gut microbial composition in a panel of both healthy and diseased individuals [16]. In general, disease status appeared to play a larger role in determining gut microbial composition than *FUT2* genotype, however, modeling both disease and secretor status together explained a larger proportion of inter-individual variation than either alone. This suggests that secretor status plays a role in determining microbiome in certain contexts. Our results were robust when considering only individuals within a healthy BMI range (a proxy for overall health) and when factoring in ancestry. However, our dataset included individuals of a wide range of ages, eating uncontrolled diets, and who may not all be healthy – all of which may mask any association of ABO and secretor status to gut microbial composition.

## Conclusions

Caveats aside, it is clear from our analysis of this large cohort that ABO and secretor status do not appear to be major drivers of microbiome composition differences across individuals. Bacterial relative abundance may differ between these groups to a small degree or may become more apparent when environmental factors such as age and diet are controlled. In addition, microbiome perturbation in disease may reveal an effect of ABO or secretor status. Regardless, these results do not support previous observations of the role of ABO or secretor status in determining microbiome composition in the gut outside of disease contexts. As the field moves forward identifying more diseases, anthropomorphic traits, and genotypes as being associated with microbiome composition, it will be important to ensure previous observations are robust by validating in large, well-powered cohorts. By doing so, the microbiome field can avoid some of the pitfalls observed from early candidate gene studies, where a myriad of associations were published that were never replicated.

## Methods

### ABO and secretor status assignment

ABO blood type data was available for 890 individuals included in the study from the TwinsUK. To increase sample size, ABO phenotype was inferred for individuals where single nucleotide polymorphism (SNP) chip genotype data was available and had been quality controlled as previously described (*n* = 1850) [29]. First, impute2 was used to phase genotypes in the region surrounding the ABO locus on chromosome 9 (positions 135000000 – 136000000, hg18), using the “-phase” option, reference mapping files from 1000 Genomes pilot + HapMap 3 release #2 (https://mathgen.stats.ox.ac.uk/impute/data_download_1000G_pilot_plus_hapmap3.html), and an effective population size of 20,000 [48]. Genotypes for three of the four SNPs described by Paré et al. were used to call ABO allele status for each phased chromosome per individual [49]. rs507666 was not genotyped in our samples and instead rs651007 was used (~4500 bp away, *r* ^*2*^ = 0.955 in CEU, see Additional file 19: Table S10). Only samples with haplotypes matching those in Additional file 19: Table S10 were retained (A1, A2, B, or O; *n* = 1503). Of these remaining samples, 763 individuals had both typed ABO status as well as genotype-inferred ABO status, and high concordance of ABO status calls were observed between the two methodologies (98% concordant). The genotype-inferred ABO status was used for those individuals who were discordant.

Secretor status was inferred for individuals with genotypes called for rs601338 in *FUT2* (*n* = 1452), where homozygosity for a G to A nonsense mutation leads to the non-secretor phenotype. Previous studies saw 100% concordance between variation in rs601338 and secretor status, as measured by a hemagglutination assay [27].

### Microbiome data collection and processing

~2000 fecal samples have been collected from the TwinsUK cohort and processed as described previously [1, 50]. Briefly, the V4 hypervariable region of the 16S gene was amplified (using primers 515F and 806R), purified, and pooled before being sequenced using the Illumina Miseq platform (2x250 paired-end sequencing). QIIME 1.8.0 [51] was used for demultiplexing, paired-end merging using fastq-join (minimum overlap 200 base pairs) [52], and quality filtering of sequence reads, including removing sequences containing uncorrectable barcodes, any ambiguous bases, or low quality reads (Phred quality scores ≤ 25). Open reference OTU clustering to Greengenes v13_8 taxonomy was performed via UCLUST at 97% sequence similarity [53]. In addition to OTU level relative abundances, read counts were collapsed by taxonomy to generate relative abundance measurements along the phylogenic tree, from genus to phylum. Unweighted UniFrac, weighted UniFrac [54], and Bray-Curtis dissimilarity [33] were calculated using an OTU table rarefied to 10,000 sequences per sample. Alpha diversity metrics (Faith’s phylogenetic diversity [55], number of observed species, Gini index [56], Chao1 richness estimator [57], and Shannon diversity [58]) were calculated from 100 rarefactions to 10,000 sequences per sample.

#### All OTU data

For analyses where all OTUs are considered, OTU relative abundances were determined by rarifying sequence data to 10,000 sequences per sample. Any OTUs not observed with at least one count across the 1503 individuals considered within this study were eliminated, leaving 88,166 OTUs. Further filtering of this OTU table was done for each analysis, as described below.

#### Common OTUs and collapsed taxa

For analyses where common taxa were considered, any OTU or collapsed taxonomic group that was not represented by at least one count across 50% of individuals the larger set of ~2000 samples was eliminated. Counts were then Box-Cox transformed using the equation:

$$ {\mathrm{y}}^{\left(\uplambda \right)} = \left({\mathrm{y}}^{\uplambda}\hbox{--}\ 1\right)/\ \uplambda $$

λ was optimized using the PowerTransform command implemented in the R package ‘car’ and an offset of one was added to handle zero counts. Relevant technical covariates (including number of 16S rRNA gene sequences per sample, age, sex, shipment date, and technician performing DNA extraction) were regressed out of transformed relative abundances. The resulting table was then trimmed to include only individuals for which inferred ABO data was available and contained information for 13 phyla, 16 classes, 20 orders, 44 families, 73 genera, and 865 OTUs – for a total of 1031 taxa considered.

### Ordination

Principal coordinates analysis (PCoA) was performed on beta-diversity metrics using the cmdscale function in R, specifying n – 1 dimensions (where n is the number of individuals for each analysis). Principal components analysis (PCA) on i) relative abundance data (residuals) of the 865 OTUs from the “common OTUs and collapsed taxa” table and ii) presence/absence calls for the OTUs represented in “all OTU data” was performed using the prcomp function in R. The top 100 PCs for all PCA and PCoA analyses were regressed against ABO status, secretor status, or ABO status only in secretors using a linear model to identify associated PCs (Additional file 3: Table S2).

Discriminant analysis of principal components (DAPC) was run using the dapc function of adegenet package in R [31] considering groups based on ABO status (all individuals), secretor status, and ABO status only in secretor individuals. For each analysis, the number of axes retained in the discriminant analysis was set to 100 and the number of axes retained in the PCA was initially set to the maximum number of possible PCs to retain (n – 1, where n is the number of individuals for each analysis). Then, the optimum number of PCs to retain was determined by the function optim.a.score and DAPC was rerun using the optimum number of PCs. A 5-fold cross-validation approach was used to assess the accuracy of DAPC to predict ABO/secretor status given input microbiome data. To determine if this cross-validation accuracy was higher than random chance, ABO/secretor status labels were permuted 100 times while maintaining twin-relationships (see below) and 5-fold cross validation run on each set of permuted data. The average accuracy across the 5 folds for each of the 100 permutations was used to determine a null distribution of accuracies. An empirical p-value was calculated by dividing the number of permutations with mean accuracies as high or higher than the actual mean accuracy by the total number of permutations.

### Permuting phenotype data while maintaining twin relationships

For several analyses, permutations were performed to determine empirical p-values, which take into account the twin status of individuals in the study. To do so, the full dataset was divided into groups of monozygotic twin pairs, dizygotic twin pairs, and unrelated individuals. For each permutation, phenotypes of unrelated individuals were randomly re-assigned within the list of unrelated individuals. For MZ and DZ twins, family IDs were randomly shuffled for each pair of twins within the two groups separately. Phenotypes were then reassigned based on family ID, so that twin structure was maintained (for example: ABO phenotypes for one pair of monozygotic twins were randomly assigned to another pair of monozygotic twins).

### Bacterial associations to ABO or secretor status

#### Linear mixed models

To determine whether the relative abundances of the 1031 common OTUs and collapsed taxa were associated with ABO or secretor status, nine different linear mixed models were run using lme4 in R [59]. Each model [1–9] below included specified fixed effects for ABO and/or secretor status and random effects for family and twin structure. Significance was assessed by comparing a full model that incorporated the model-specific fixed effects to a reduced model, which only included the random effects using the anova function in R. q-values were used to account for multiple testing within each model separately [60]. The models, specified in R/lme4 notation, included:

- [m1] ***y***_***i***_ = *β*_*0*_ + *β*_*1*_ **A**_i_ + *β*_*2*_ **AB**_i_ + *β*_*3*_ **B**_i_ + (1|**FAM**_i_) + (1|**MZID**_i_) + ε
- [m2] ***y***_*i*_ = *β*_*0*_ + *β*_*1*_ **O**_i_ + (1|**FAM**_i_) + (1|**MZID**_i_) + ε
- [m3] ***y***_*i*_ = *β*_*0*_ + *β*_*1*_ **B**_i_ + (1|**FAM**_**i**_) + (1|**MZID**_**i**_) + ε
- [m4] ***y***_*i*_ = *β*_*0*_ + *β*_*1*_ **(B or AB)**_i_ + (1|**FAM**_**i**_) + (1|**MZID**_**i**_) + ε
- [m5] ***y***_*i*_ = *β*_*0*_ + *β*_*1*_ **SS**_i_ + (1|**FAM**_**i**_) + (1|**MZID**_**i**_) + ε
- [m6] same as model 1, but in secretor individuals only
- [m7] same as model 3, but in secretor individuals only
- [m8] ***y***_***i***_ = *β*_*0*_ + *β*_*1*_ **A**_i_ + *β*_*2*_ **AB**_i_ + *β*_*3*_ **B**_i_ + *β*_*4*_ **SS**_i_ + *β*_*5*_ **SS**_i_*A_i_ + *β*_*6*_ **SS**_i_*AB_i_ + *β*_*7*_ **SS**_i_*B_i_ + (1|**FAM**_i_) + (1|**MZID**_i_) + ε
- [m9] ***y***_***i***_ = *β*_*0*_ + *β*_*1*_ **B**_i_ + *β*_*2*_ **SS**_i_ + *β*_*3*_ **SS**_i_*B_i_ + (1|**FAM**_i_) + (1|**MZID**_i_) + ε

where ***y***_*i*_ is the residual relative abundance of one of the common OTUs or collapse taxa, fixed effects for ABO status (**A**_i_ takes the value 1 if individual i is A, **AB**_i_ take the value 1 if individual i is AB, **B**_i_ takes the value 1 if individual i is B, **O**_i_ takes the value 1 if individual i is O) and secretor status (**SS**_i_ takes the value 1 if an individual is a secretor) are specific to the given model, random effects account for family (1|**FAM**_i_) and increased genetic sharing of monozygotic twins (1|**MZID**_i_), and residual error ε is assumed *N*(0,σ_ε_^2^) (Additional file 8: Table S3).

Variants in the lactase gene (*LCT*) have been associated with relative abundance of Bifidobacteria in the gut [2, 35]. To account for this potential confounder, models [m1-m9] listed above were rerun for the 6 common OTUs that classify to the *Bifidobacterium* genus and the collapsed taxonomies for genus *Bifidobacterium*, family Bifidobacteriaceae, and order Bifidobacteriales, incorporating an additional fixed effect for rs1446585, a SNP in the *LCT* gene that is associated with Bifidobacterium relative abundance in the TwinsUK cohort. *P*-values were determined as stated above and q-values were used to take into account multiple tests (Additional file 13: Table S6).

#### Presence/absence associations

In addition to testing whether relative abundances of the common OTUs and collapsed taxonomic groups were associated to ABO or secretor status, the presence/absence of a wider range of OTUs was also examined. To do so, any OTUs from the rarefied OTU table (described above) that were not observed in at least 10% of individuals were discarded, leaving 1692 OTUs. Relative abundances of these OTUs were transformed into 1/0 for presence or absence for each individual, and Chi-squared tests of independence were run using the chisq.test in R for each OTU for ABO status, secretor status, and ABO status in secretors only. *P*-values were determined by a permutation scheme with 1000 permutations that took twin status into account (see above). q-values were used to correct for multiple testing (Additional file 12: Table S5) [60].

### Correcting for ancestry using principal component analysis

Both genetic and cultural factors related to ancestry could potentially influence microbial composition in the gut, masking associations of the microbiota with ABO or secretor status. To take ancestry into account, models 1–9 above were rerun including genetic principal components as fixed effects. First, genome-wide SNP data (as described in Goodrich et al. [35]) were filtered to remove any variants with a minor allele frequency less than 5% or Hardy-Weinberg equilibrium P-values less than 0.001 in the individuals included in this study (*n* = 1493). Then, SNPs were pruned for linkage disequilibrium using a window of 1000 kb, a step size of 50 SNPs, and a pairwise *r* ^*2*^ threshold of 0.2 in plink1.9 [61]. Finally, smartpca [62] was used to perform principal components analysis on the remaining 74,418 SNPs. The top five principal components explain 95.1% of the genetic variation in the dataset and were included as fixed effect terms in linear models 1–9 to control for ancestry.

### Diversity analyses

#### ABO/secretor status associations with alpha diversity metrics

Linear mixed models were used to assess whether there were significant diversity differences according to ABO or secretor status. First, relevant covariates were regressed out of alpha diversity metrics (including number of 16S rRNA gene sequences per sample, age, sex, shipment date, and technician performing DNA extraction). Then, association of the residuals to ABO, secretor status, and ABO status in secretors only were assessed by linear mixed models parameterized as described in models [m1], [m5], and [m6] (Fig. 2, Additional file 5: Figure S3). Additionally, within each ABO class (A, B, AB, and O individuals), alpha diversity metric differences were compared between secretors and non-secretors using linear mixed models with a fixed effect term for secretor status and random effect terms for family and zygosity as described above (Additional file 6: Figure S4).

#### Beta-diversity comparisons between concordant and discordant individuals

To assess whether individuals who shared the same ABO or secretor status also tended to have more similar microbiomes, average beta diversity (unweighted UniFrac, weighted UniFrac, and Bray Curtis dissimilarity) was compared between sets of individuals concordant and discordant for the phenotype of interest. Comparisons were made using i) all individuals and ii) one twin per family to eliminate the potential bias of shared genetics and environment expected between sets of twins. Significance was assessed using a permutation strategy (1000 permutations) that took into account the twin relationships in the data, as described above. Additionally, permutation analyses confirmed that pairs of twins in general have more similar microbiomes than pairs of unrelated individuals (family ID was permuted across all individuals 1000 times to generate null distribution) and that pairs of MZ twins have more similar microbiomes that DZ twins (the zygosity labels were permuted 1000 times across all twin pairs to generate null distribution).

### Joint modeling approaches

In addition to identifying individual taxa that may be associated with ABO or secretor status, two separate approaches were used to jointly model the relative abundances of multiple taxa simultaneously.

#### Random forests

This method was implemented using the randomForest package with R, setting the number of trees to grow to 500. Imbalanced group sizes reduce effectiveness of random forests [42]. To compensate for this potential issue in the data, the random forests analysis was rerun, but groups were down-sampled to the smallest group size within each analysis by setting the sampsize parameter in the randomForest function.

#### MGSDA

In addition to random forests, multi-group sparse discriminant analysis (MGSDA) methodology was applied to identify bacterial taxa that in combination can predict ABO or secretor status [43]. Variable selection and classification was performed using the MGSDA package in R and 5-fold cross-validation was applied to assess classification accuracy for three different models using two datasets. The three models included dependent variables for i) ABO status, ii) secretor status, and iii) ABO status only in secretors. The first data set included all common OTUs and taxa, while the second included the rarefied relative abundances of all OTUs present in at least 10% of individuals (*n* = 1692 OTUs).

## Additional files

Additional file 1: Table S1. Cohort characteristics in the full and ‘healthy’ datasets. The total nubmer (n) or percent of total (%) for each category is listed for either the full or ‘healthy’ datasets, along with the standard deviation for each measure. (XLSX 36 kb)
Additional file 2: Figure S1. ABO and secretor statuses are not associated with broad compositional differences through PCoA. In addition to examining PCoA of unweighted UniFrac distance (Fig. 1), Bray Curtis dissimilarity and weighted UniFrac distance was used to determine if there were broad compositional differences in the microbiome between ABO or secretor groups. The first two principal coordinates are displayed for Bray Curtis dissimilarity (A, C, E) and weighted UniFrac distance (B, D, F) along the *x*- and *y*-axes. Points are colored by ABO status (A, B), secretor status (C, D), and ABO status in secreting individuals only (E, F). None of the top 100 principal coordinates are significantly associated with ABO or secretor status for any beta-diversity metric examined. (PDF 135 kb)
Additional file 3: Table S2. P- and q-values for PCA. This spreadsheet contains the *p*-values and q-values for associations of the top 100 PCs to ABO and secretor status consider 5 different ordination techniques (a separate tab for each). The ordination method for each table and the column description within each table are described in the document. (XLSX 57 kb)
Additional file 4: Figure S2. ABO and secretor phenotypes are not associated with broad compositional differences through PCA. Principal components analysis of the presence/absence of all OTUs in at least 10% of individuals in the TwinsUK dataset does not reveal any significant associations between the top 100 PCs and ABO or secretor status. The first two principal components (PCs) are displayed along the *x*- and *y*-axes. Points are colored by ABO status (A), secretor status (B), or ABO status in secreting individuals only (C). (PDF 102 kb)
Additional file 5: Figure S3. Alpha diversity does not significantly differ according to ABO or secretor status. In addition to Faith‘s phylogenic diversity (Fig. 2), other alpha diversity metrics do not significantly differ according to ABO status (A-D), secretor status (E-H), or ABO status in secreting individuals only (I-L) as determined by linear mixed models (*P* > 0.05). Alpha metrics considered included: (A, E, I) The number of observed species; (B, F, J) the Chao 1 richness estimator, which estimates the actual number of species in a community, as doing a microbial census through sequencing will likely not sample all rare members; (C, G, K) the Gini coefficient, which measures community evenness; (D, H, L) the Shannon diversity index, which accounts or both the number of taxa as well as their abundance within a sample. (PDF 182 kb)
Additional file 6: Figure S4. Alpha diversity differs significantly between secretors and non-secretors for AB and B individuals. While there are no significant differences between secretors and non-secretors overall, secreting AB individuals have more diverse microbiomes and secreting B individuals have less diverse microbiomes than non-secreting AB or B individuals, respectively (for all alpha diversity metrics except Shannon diversity (E)). It is important to note that the AB (*n* = 40) and B (*n* = 140) groups of individuals are smaller than the A (*n* = 606) and O (*n* = 717) groups. Significance codes: not significant = NS, *P* ≤ 0.05 = *. *x*-axis abbreviations: non-secretor = NS, secretor = S. (PDF 99 kb)
Additional file 7: Figure S5. Microbiomes are not more similar for pairs of individuals concordant for either ABO or secretor status compared to discordant pairs. In addition to unweighted UniFrac distance, pairwise similarity of the microbiome was assessed using weighted UniFrac distance (A) and Bray Curtis dissimilarity (B). As in Fig. 2, pairs of related individuals in general have more similar microbiomes than pairs of unrelated individuals, and monozygotic twins have more similar microbiomes than dizygotic twins, pointing to host genetic control of the microbiome. However, when beta-diversity is stratified by either ABO or secretor status, no significant differences are observed between pairs of individuals concordant for status compared to individuals discordant for status. “All individuals” includes all pairs of individuals in the dataset, including pairs of twins. To ensure family relationships did not lead to bias in beta-diversity, one twin from each twin pair was removed for “one twin per family”. The number of pairwise comparisons in each category is displayed (“*n* = “). Significance codes: not significant = NS, *P* ≤ 0.05 = *, *P* ≤ 0.01 = **, *P* ≤ 0.001 = ***, *P* ≤ 0.0001 = ****. (PDF 34 kb)
Additional file 8: Table S3. P- and q-values for linear mixed models. This table contains the *p*-values and q-values for the linear mixed models relating the abundances of the common OTUs and collapsed taxa to ABO and secretor status. Models 1-9 are described fully in the methods section, but a brief description of each is included in the document. *P*-values were calculated by comparing a full model to a reduced model, which consisted of only the random effects for family and twin status. Q-values were calculated within each model separately. (XLSX 236 kb)
Additional file 9: Figure S6. QQ-plot of the 9 linear mixed models comparing ABO and secretor phenotypes to taxon relative abundance. Expected –log_10_(*P*-values) are plotted (*x*-axis) compared to the observed –log_10_(*P*-values) along the *y*-axis. Tests significant at *q* ≤ 0.1 and *q* ≤ 0.05 thresholds are indicated with larger point sizes for linear mixed models 1-9 (A-E). Model descriptions can be found in the methods section. (PDF 103 kb)
Additional file 10: Figure S7. QQ-plot of the 9 linear mixed models comparing ABO and secretor phenotypes to taxon relative abundance, including genetic ancestry in the model. Expected –log_10_(*P*-values) are plotted (*x*-axis) compared to the observed –log_10_(*P*-values) along the *y*-axis. Tests significant at *q* ≤ 0.1 and *q* ≤ 0.05 thresholds are indicated with larger point sizes for linear mixed models 1-9 (A-E). Model descriptions can be found in the methods section. (PDF 103 kb)
Additional file 11: Table S4. P- and q-values for linear mixed models, controlling for ancestry. This table contains the *p*-values and q-values for the linear mixed models relating the abundances of the common OTUs and collapsed taxa to ABO and secretor status. Models 1-9 are described fully in the methods section, but a brief description of each is included below. These models also include the top 5 principal component loadings as fixed effects to control for ancestry. *P*-values were calculated by comparing a full model to a reduced model, which consisted of only the top 5 PC loadings (as fixed effects) and the random effects for family and twin status. Q-values were calculated within each model separately. (XLSX 237 kb)
Additional file 12: Table S5. P- and q-values for Chi-squared tests of independence. This supplemental file contains the *p*-values and q-values for Chi-squared tests of independence according to ABO status, secretor status, or ABO status in secreting individuals only. Tests were conducted for the presence/absence of any OTU that had at least one count in 10% of individuals after rarifying total sequencing depth to 10,000 reads. Explanations for each tab and column in each tab are listed in the document. (XLSX 299 kb)
Additional file 13: Table S6. P- and q-values for linear mixed models for *Bifidobacteria*, including *LCT allele* status in the model. This table contains the *p*-values and Benjamini-Hochberg adjusted *p*-values for the linear mixed models relating the abundances of the common Bifidobacterium OTUs and collapsed taxa to ABO and secretor status, while including LCT variation in the model (rs1446585 genotype). Models 1-9 are described fully in the methods section, but a brief description of each is included below. *P*-values were calculated by comparing a full model to a reduced model, which consisted of only the random effects for family and twin status and a fixed effect for *LCT allele* status. P-values were adjusted within each model separately. Descriptions of each column are included in the document. (XLSX 29 kb)
Additional file 14: Figure S8. The microbiome is not able to classify ABO or secretor status with high accuracy through random forests. Confusion matrices list the total number samples from a given sample class (*x*-axis) classified into each predicted class (*y*-axes) from six random forests models. The total out-of-bag (OOB) error is indicated above each confusion matrix. The total number of individuals (“*n* = “) and the error of classification (as a %) for each class are listed below the *x*-axis. Random forests was run to classify samples based on ABO status (A, B), secretor status (C, D), and ABO status in secretors (E, F). Two implementation methodologies were considered: first, all samples were included for the tree building process (A, C, E – unbalanced model). Uneven group sizes can lead to the majority group being overrepresented in predictions in random forests, as is observed in our data. To address this issue, a second implementation down-sampled groups to the smallest group size in each test (B, D, F – balanced model). Error rates of all models were high 27–66%), therefore, relative abundances of the most common microbiota are not able to predict ABO or secretor status accurately. (PDF 126 kb)
Additional file 15: Table S7. MGSDA classification accuracy. This table contains tables of accuracy as determined by 5-fold cross validation for MGSDA. MGSDA was run using the covariate-corrected, transformed abundances of all common OTUs and taxa (table “common_taxa”). Additionally, MGSDA was run on the abundances of OTUs present in at least 10% of individuals in the dataset (table “OTUs_in_10_percent”). The groups considered in MGSDA with each data set were ABO status, secretor status, and ABO status in secreting individuals only, which are indicated as columns in each tab. A description of what is represented in each row is included in the document. (XLSX 30 kb)
Additional file 16: Table S8. Featured identified by MGSDA. This supplemental file contains the features identified by MGSDA as being predictive of ABO status, secretor status, or ABO status in secretors. Each tab contains the feature identified (may be an OTU or collapsed taxonomic identification) as well as the taxonomy (relevant for OTUs). Each tab contains the results for a different implementation of MGSDA, described further in the document. (XLSX 44 kb)
Additional file 17: Figure S9. Analyses using only individuals with BMI < 25 recapitulate results. A-C) Neither ABO or secretor status associated with broad compositional differences of the gut microbiota in the TwinsUK. None of the top 100 principal coordinates (PCs) from principal coordinate analysis of unweighted UniFrac distance are significantly associated with either ABO or secretor status. The first two PCs are shown, colored by ABO status (A) and secretor status (B). (C) Discriminant analysis of PCA (DAPC) is largely unsuccessful at predicting ABO or secretor status from microbiome data. The mean accuracy from 5-fold cross validation is plotted for ABO status, secretor status, and ABO status only in secreting individuals (yellow). Significance was determined by comparing the accuracy of each test to the accuracies of permuted data, which took into account twin relationships (gray). D-F) Microbiome diversity does not significantly differ by ABO, but does by secretor status. Within sample diversity (Faith’s phylogenic diversity) is significantly different between secretors versus non-secretors (D, *P* < 0.05), but not across the ABO groups in all individuals (E, *P* > 0.05), or across ABO groups in only secreting individuals (C, *P* > 0.05). (F) Microbiomes are more similar for siblings versus pairs of unrelated individuals, as measured by unweighted UniFrac distance. However, microbiomes of pairs of individuals concordant for either ABO or secretor status are not more similar than for pairs of individuals who are discordant. This holds true when all individuals in the dataset are considered (“all individuals”) or when only one individual from each twin pair is examined (“one twin per family”). The total number of pairs of individuals within each boxplot is indicated with “*n* = “. H) None of the common taxa are associated with ABO or secretor status. QQ-plot displaying the expected –log_10_(*P*-value) compared to the –log_10_(*P*-value) for all taxa tested in linear mixed models 6 (light gray points) and 8 (dark gray points, as plotting in Fig. 3). Significance codes: *P* ≤ 0.05 = *, *P* ≤ 0.01 = **, *P* ≤ 0.001 = ***, *P* ≤ 0.0001 = ****, not significant = NS. (PDF 387 kb)
Additional file 18: Table S9. P- and q-values for linear mixed models, only including individuals with BMI <  25. This table contains the *p*-values and q-values for the linear mixed models relating the abundances of the common OTUs and collapsed taxa to ABO and secretor status, for only individuals with a BMI < = 25. Models 1-9 are described fully in the methods section, but a brief description of each is included below. *P*-values were calculated by comparing a full model to a reduced model, which consisted of only the random effects for family and twin status. Q-values were calculated within each model separately. Descriptions of each column are included in the document. (XLSX 229 kb)
Additional file 19: Table S10. ABO alleles in the TwinsUK cohort. The phased SNPs used to call ABO status are listed, along with the haplotype count and frequency within the TwinsUK samples. (XLSX 30 kb)

## Abbreviations

DAPC: Discriminant analysis of PCA
DGGE: Denaturing gradient gel electrophoresis
DZ: Dizygotic
MGSDA: Multi-group sparse discriminant analysis
MZ: Monozygotic
OOB: Out-of-bag
OTU: Operational taxonomic unit
PC: Principal component or coordinate
PCA: Principal component analysis
PCoA: Principal coordinate analysis
SNP: Single nucleotide polymorphism
TwinsUK: United Kingdom Adult Twin Health Registry
