# Supplementary material for: ABO antigen and secretor statuses are not associated with gut microbiota composition in 1,500 twins
Source: BMC Genomics. 2016 Nov 21;17:941. doi: 10.1186/s12864-016-3290-1 (PMC5117602; doi:10.1186/s12864-016-3290-1)
Supplement: Additional file 2: Figure S1. — ABO and secretor statuses are not associated with broad compositional differences through PCoA. In addition to examining PCoA of unweighted UniFrac distance (Fig. 1), Bray Curtis dissimilarity and weighted UniFrac distance was used to determine if there were broad compositional differences in the microbiome between ABO or secretor groups. The first two principal coordinates are displayed for Bray Curtis dissimilarity (A, C, E) and weighted UniFrac distance (B, D, F) along the x- and y-axes. Points are colored by ABO status (A, B), secretor status (C, D), and ABO status in secreting individuals only (E, F). None of the top 100 principal coordinates are significantly associated with ABO or secretor status for any beta-diversity metric examined. (PDF 135 kb) [file 12864_2016_3290_MOESM2_ESM.pdf]

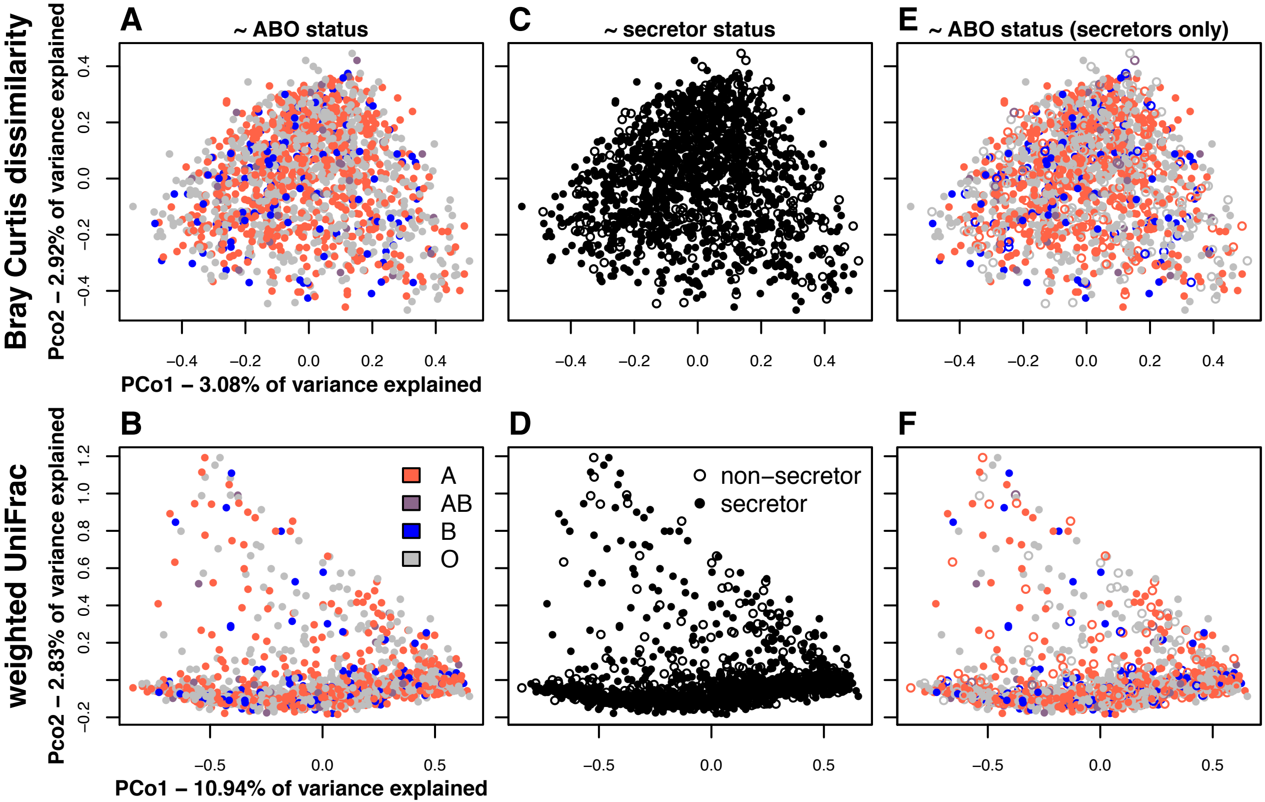

Bray Curtis dissimilarity
weighted UniFrac
A
~ ABO status
C
~ secretor status
E
~ ABO status (secretors only)
B
D
F
Pco2 – 2.92% of variance explained
PCo1 – 3.08% of variance explained
Pco2 – 2.83% of variance explained
PCo1 – 10.94% of variance explained
A
AB
B
O
non–secretor
secretor
−0.4
−0.2
0.0
0.2
0.4
−0.5
0.0
0.5
1.2
1.0
0.8
0.6
