# Supplementary figures and images for: ABO antigen and secretor statuses are not associated with gut microbiota composition in 1,500 twins
Source: BMC Genomics. 2016 Nov 21;17:941. doi: 10.1186/s12864-016-3290-1 (PMC5117602; doi:10.1186/s12864-016-3290-1)

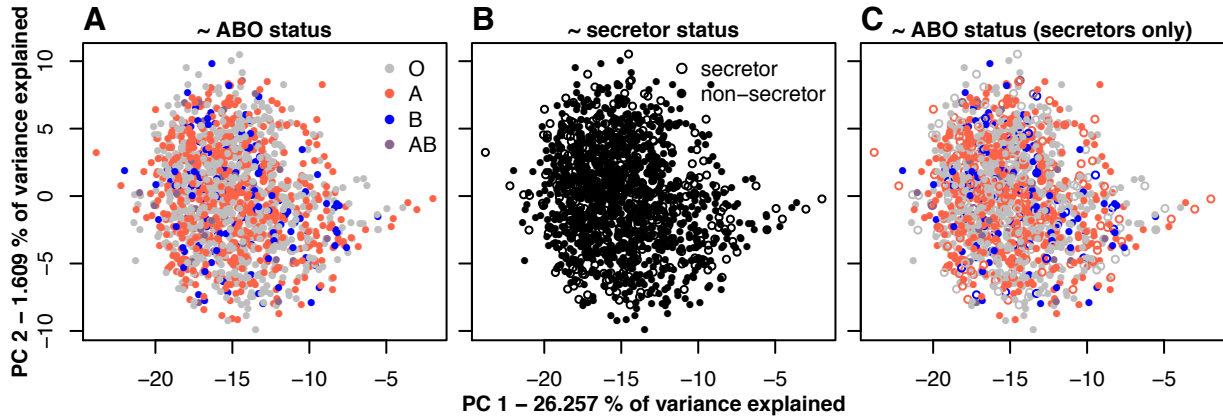

Supplement: Additional file 4: Figure S2. — ABO and secretor phenotypes are not associated with broad compositional differences through PCA. Principal components analysis of the presence/absence of all OTUs in at least 10% of individuals in the TwinsUK dataset does not reveal any significant associations between the top 100 PCs and ABO or secretor status. The first two principal components (PCs) are displayed along the x- and y-axes. Points are colored by ABO status (A), secretor status (B), or ABO status in secreting individuals only (C). (PDF 102 kb) [file 12864_2016_3290_MOESM4_ESM.pdf]

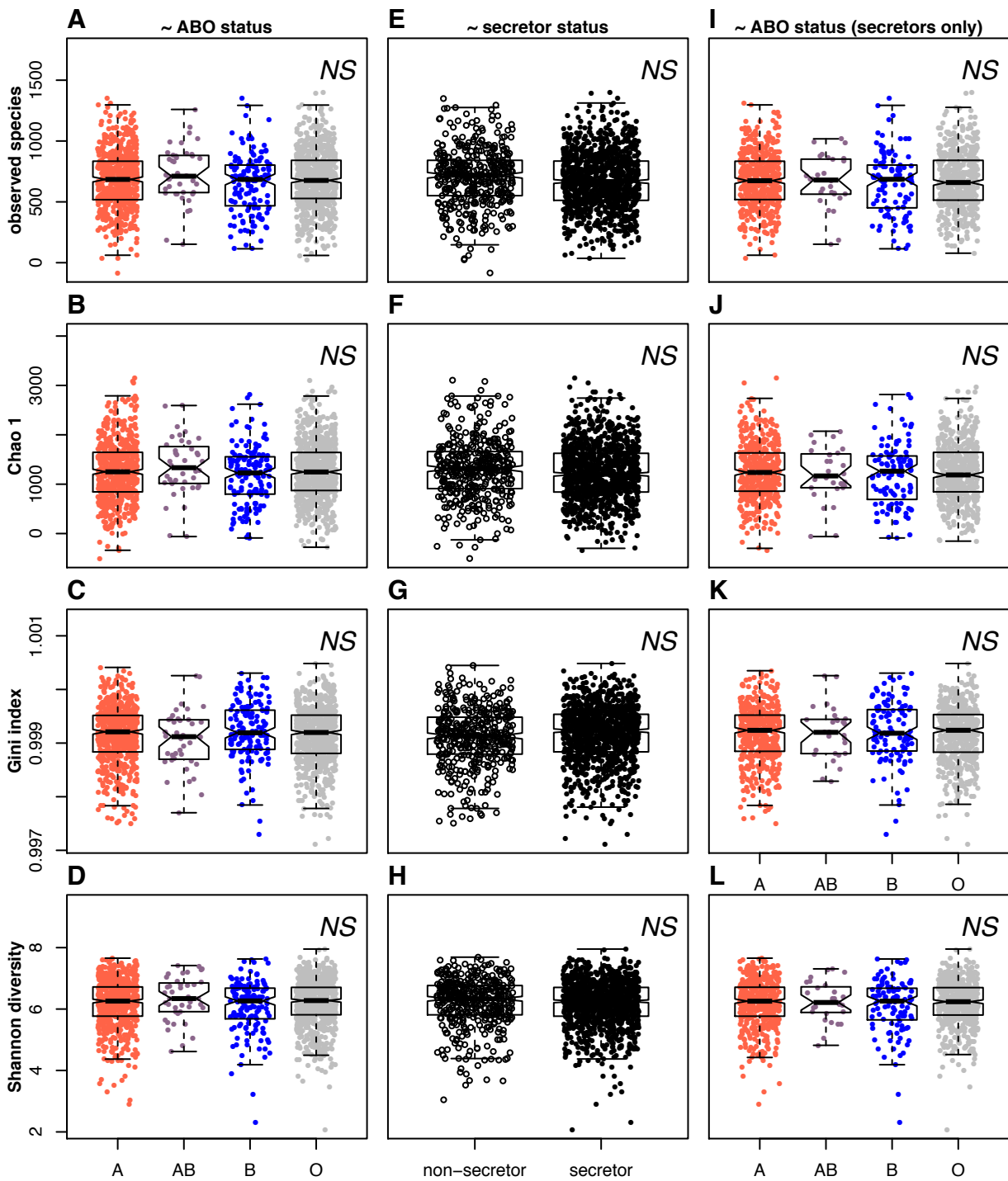

Supplement: Additional file 5: Figure S3. — Alpha diversity does not significantly differ according to ABO or secretor status. In addition to Faith‘s phylogenic diversity (Fig. 2), other alpha diversity metrics do not significantly differ according to ABO status (A-D), secretor status (E-H), or ABO status in secreting individuals only (I-L) as determined by linear mixed models (P > 0.05). Alpha metrics considered included: (A, E, I) The number of observed species; (B, F, J) the Chao 1 richness estimator, which estimates the actual number of species in a community, as doing a microbial census through sequencing will likely not sample all rare members; (C, G, K) the Gini coefficient, which measures community evenness; (D, H, L) the Shannon diversity index, which accounts or both the number of taxa as well as their abundance within a sample. (PDF 182 kb) [file 12864_2016_3290_MOESM5_ESM.pdf]

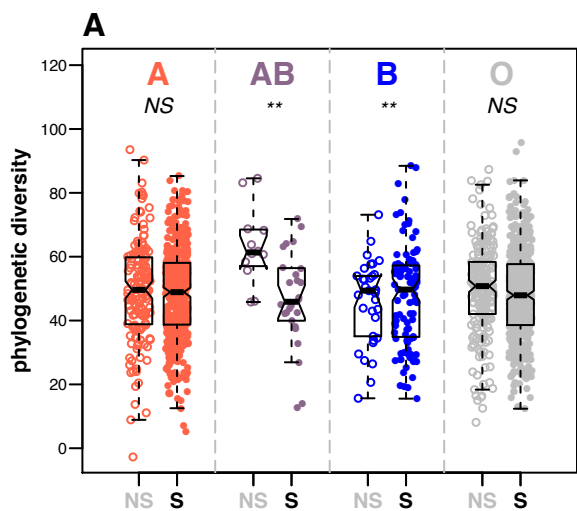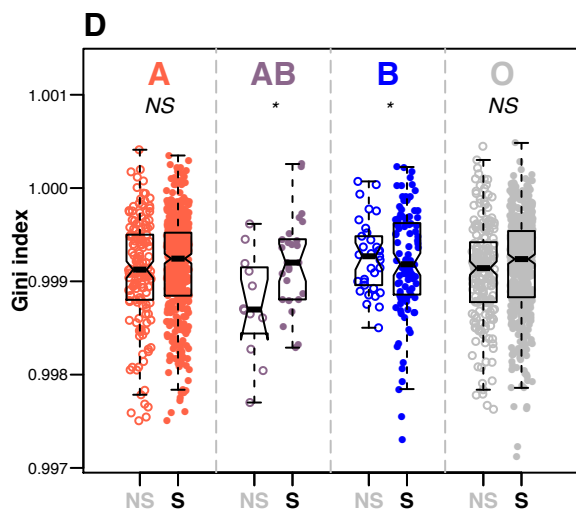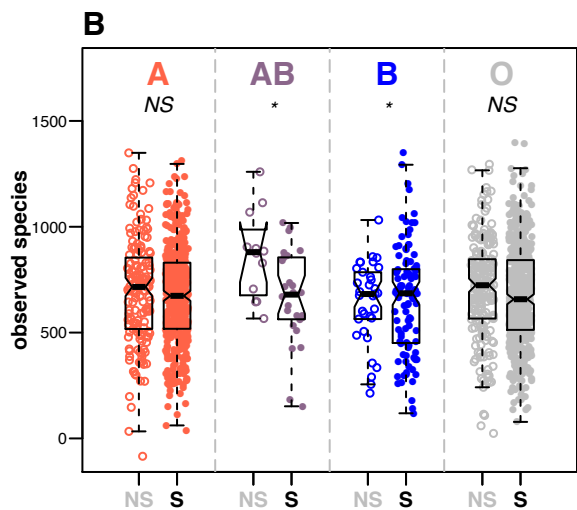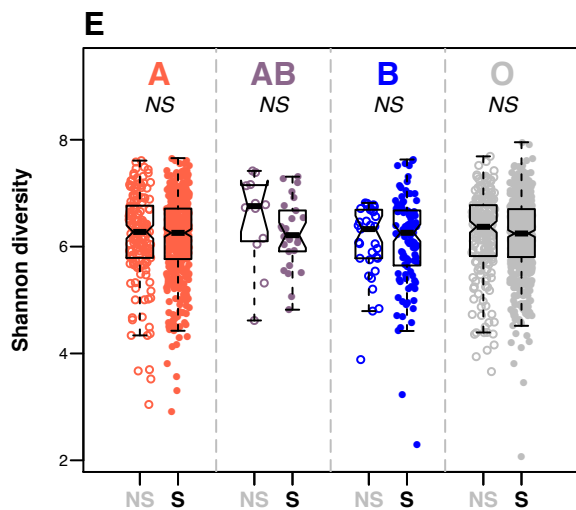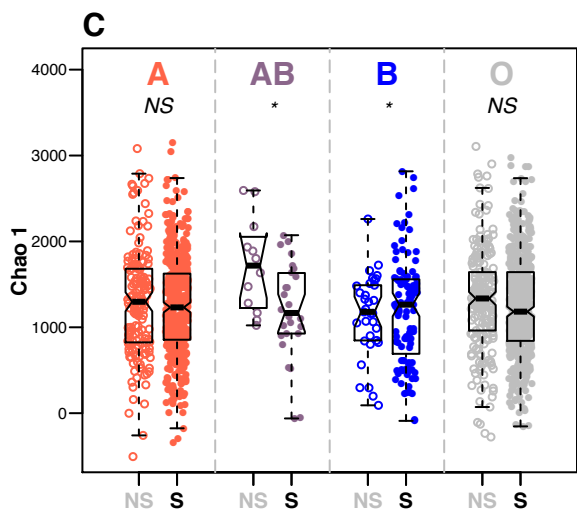

Supplement: Additional file 6: Figure S4. — Alpha diversity differs significantly between secretors and non-secretors for AB and B individuals. While there are no significant differences between secretors and non-secretors overall, secreting AB individuals have more diverse microbiomes and secreting B individuals have less diverse microbiomes than non-secreting AB or B individuals, respectively (for all alpha diversity metrics except Shannon diversity (E)). It is important to note that the AB (n = 40) and B (n = 140) groups of individuals are smaller than the A (n = 606) and O (n = 717) groups. Significance codes: not significant = NS, P ≤ 0.05 = *. x-axis abbreviations: non-secretor = NS, secretor = S. (PDF 99 kb) [file 12864_2016_3290_MOESM6_ESM.pdf]

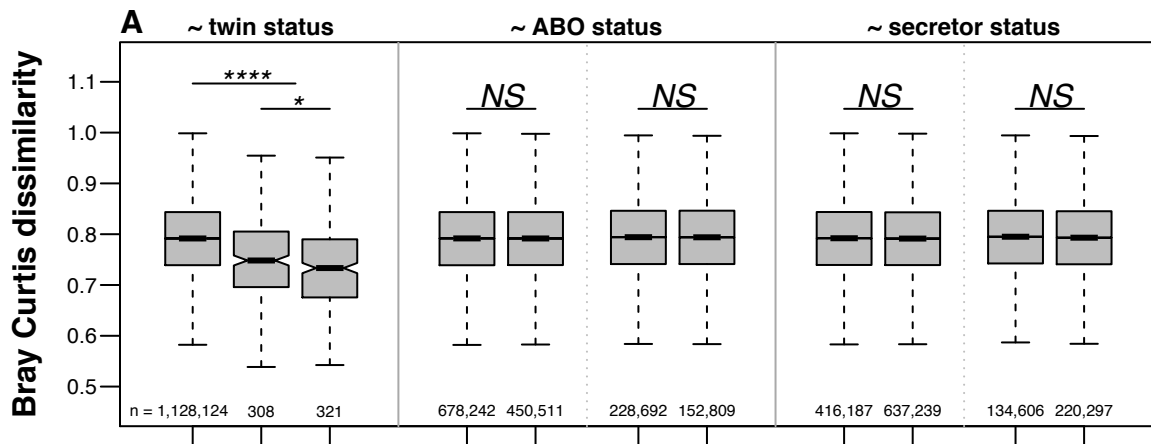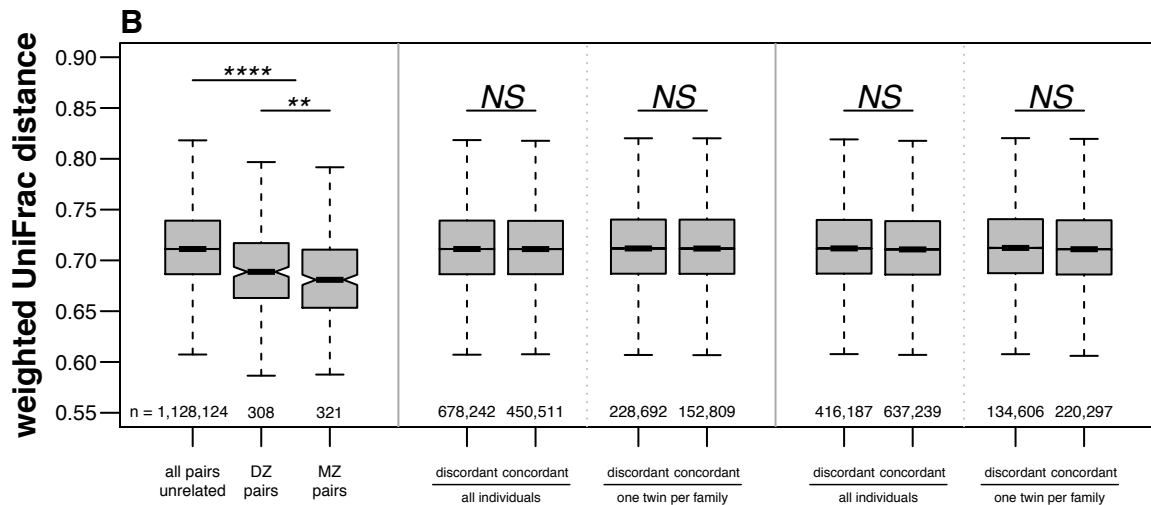

Supplement: Additional file 7: Figure S5. — Microbiomes are not more similar for pairs of individuals concordant for either ABO or secretor status compared to discordant pairs. In addition to unweighted UniFrac distance, pairwise similarity of the microbiome was assessed using weighted UniFrac distance (A) and Bray Curtis dissimilarity (B). As in Fig. 2, pairs of related individuals in general have more similar microbiomes than pairs of unrelated individuals, and monozygotic twins have more similar microbiomes than dizygotic twins, pointing to host genetic control of the microbiome. However, when beta-diversity is stratified by either ABO or secretor status, no significant differences are observed between pairs of individuals concordant for status compared to individuals discordant for status. “All individuals” includes all pairs of individuals in the dataset, including pairs of twins. To ensure family relationships did not lead to bias in beta-diversity, one twin from each twin pair was removed for “one twin per family”. The number of pairwise comparisons in each category is displayed (“n = “). Significance codes: not significant = NS, P ≤ 0.05 = *, P ≤ 0.01 = **, P ≤ 0.001 = ***, P ≤ 0.0001 = ****. (PDF 34 kb) [file 12864_2016_3290_MOESM7_ESM.pdf]

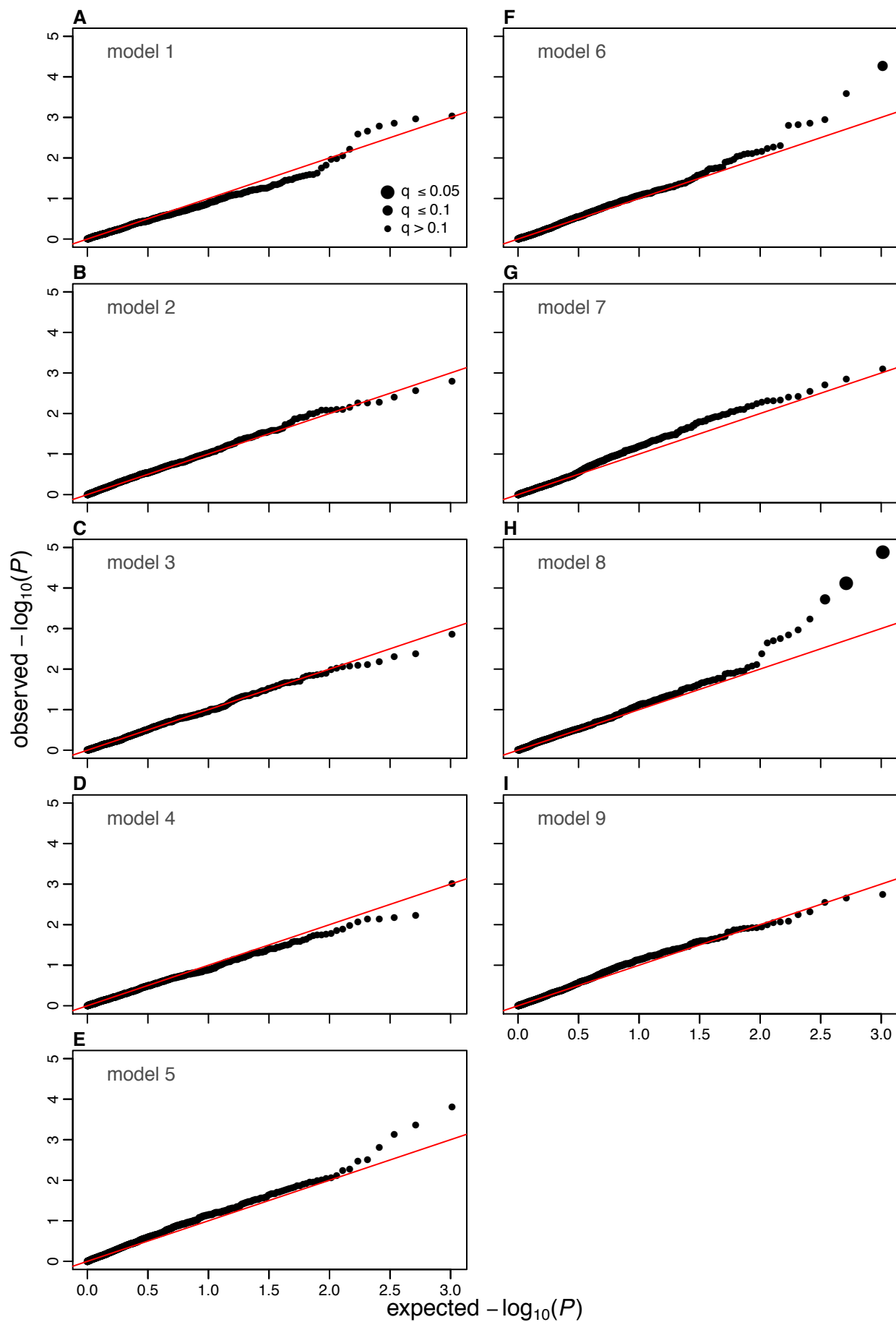

Supplement: Additional file 9: Figure S6. — QQ-plot of the 9 linear mixed models comparing ABO and secretor phenotypes to taxon relative abundance. Expected –log10(P-values) are plotted (x-axis) compared to the observed –log10(P-values) along the y-axis. Tests significant at q ≤ 0.1 and q ≤ 0.05 thresholds are indicated with larger point sizes for linear mixed models 1-9 (A-E). Model descriptions can be found in the methods section. (PDF 103 kb) [file 12864_2016_3290_MOESM9_ESM.pdf]

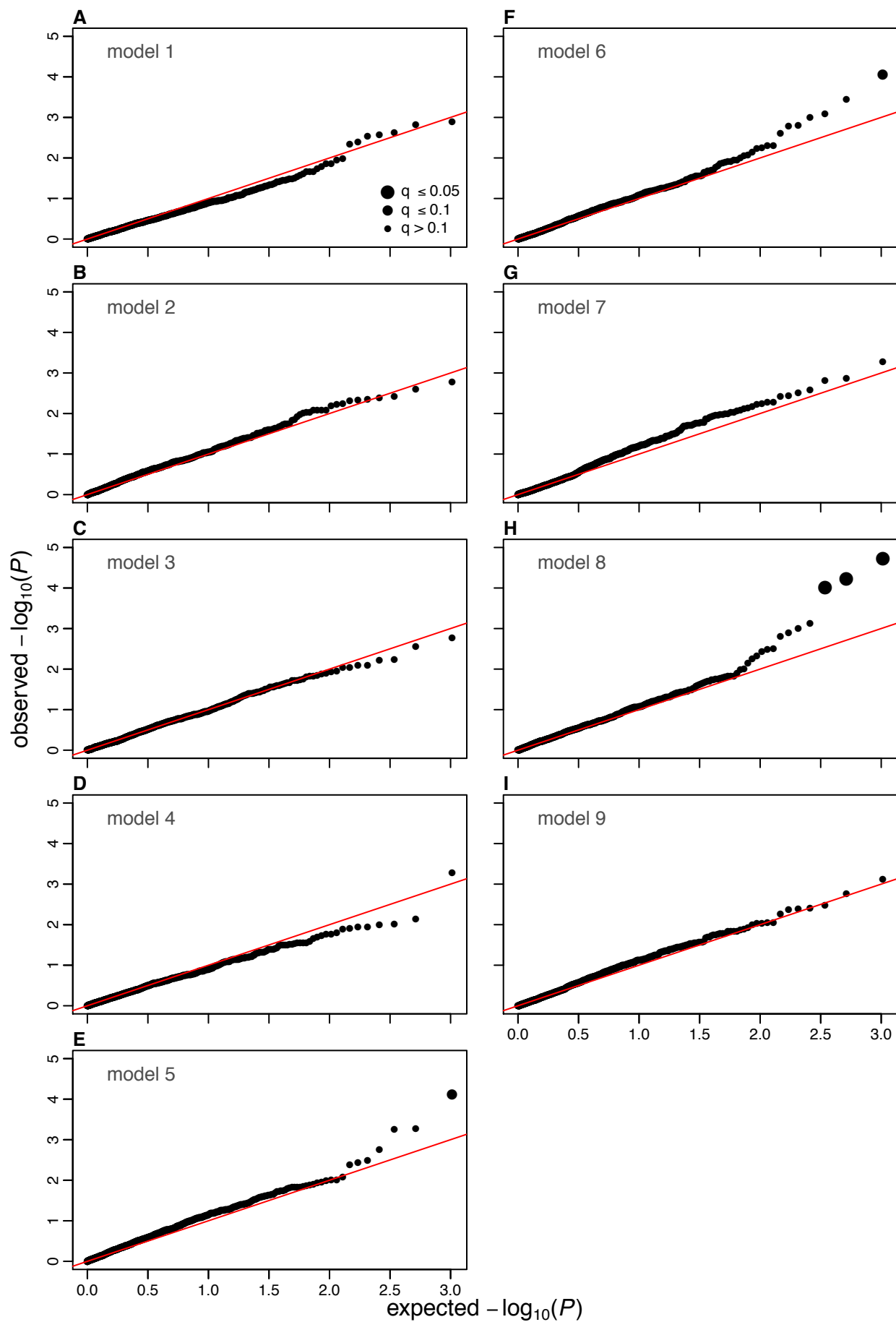

Supplement: Additional file 10: Figure S7. — QQ-plot of the 9 linear mixed models comparing ABO and secretor phenotypes to taxon relative abundance, including genetic ancestry in the model. Expected –log10(P-values) are plotted (x-axis) compared to the observed –log10(P-values) along the y-axis. Tests significant at q ≤ 0.1 and q ≤ 0.05 thresholds are indicated with larger point sizes for linear mixed models 1-9 (A-E). Model descriptions can be found in the methods section. (PDF 103 kb) [file 12864_2016_3290_MOESM10_ESM.pdf]

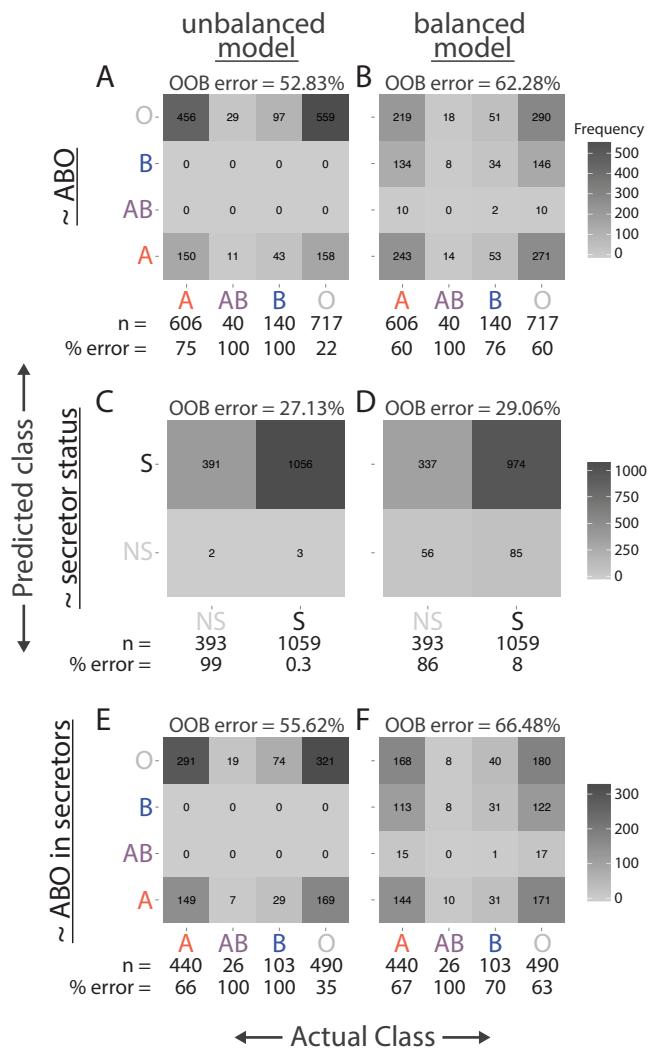

Supplement: Additional file 14: Figure S8. — The microbiome is not able to classify ABO or secretor status with high accuracy through random forests. Confusion matrices list the total number samples from a given sample class (x-axis) classified into each predicted class (y-axes) from six random forests models. The total out-of-bag (OOB) error is indicated above each confusion matrix. The total number of individuals (“n = “) and the error of classification (as a %) for each class are listed below the x-axis. Random forests was run to classify samples based on ABO status (A, B), secretor status (C, D), and ABO status in secretors (E, F). Two implementation methodologies were considered: first, all samples were included for the tree building process (A, C, E – unbalanced model). Uneven group sizes can lead to the majority group being overrepresented in predictions in random forests, as is observed in our data. To address this issue, a second implementation down-sampled groups to the smallest group size in each test (B, D, F – balanced model). Error rates of all models were high 27–66%), therefore, relative abundances of the most common microbiota are not able to predict ABO or secretor status accurately. (PDF 126 kb) [file 12864_2016_3290_MOESM14_ESM.pdf]

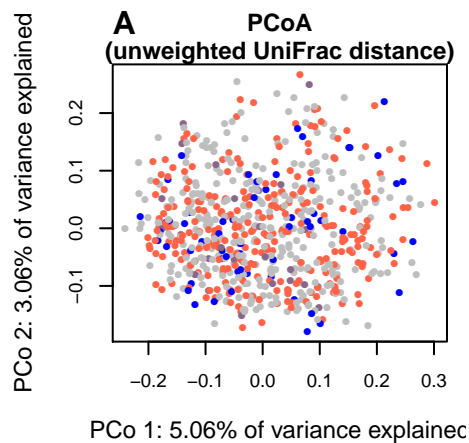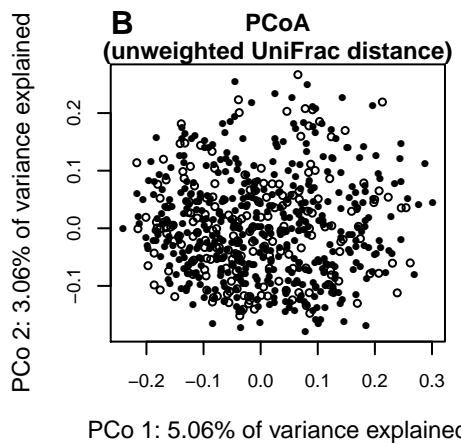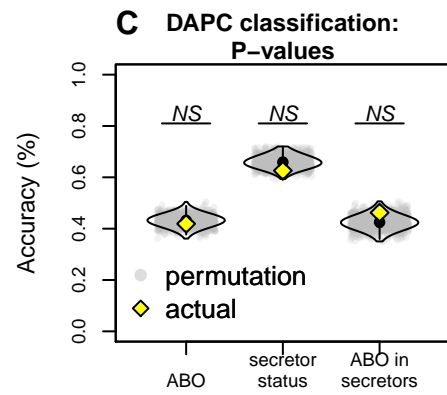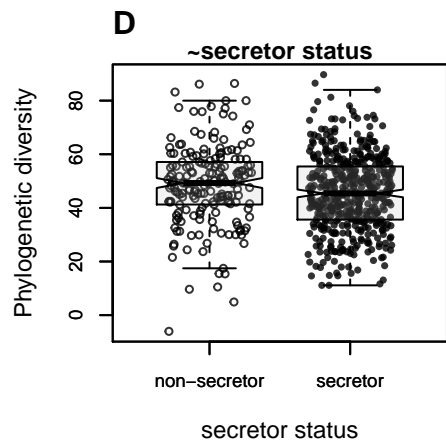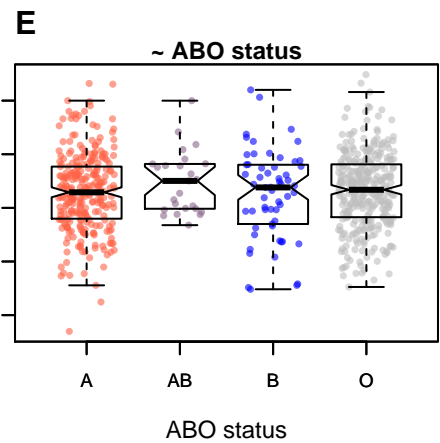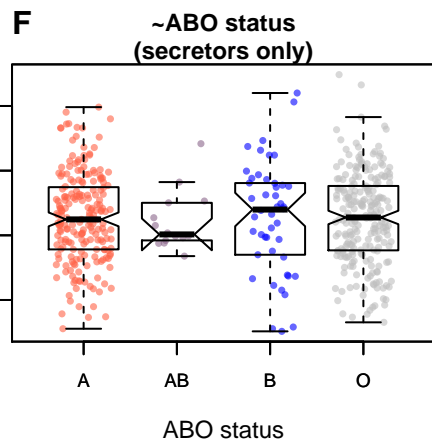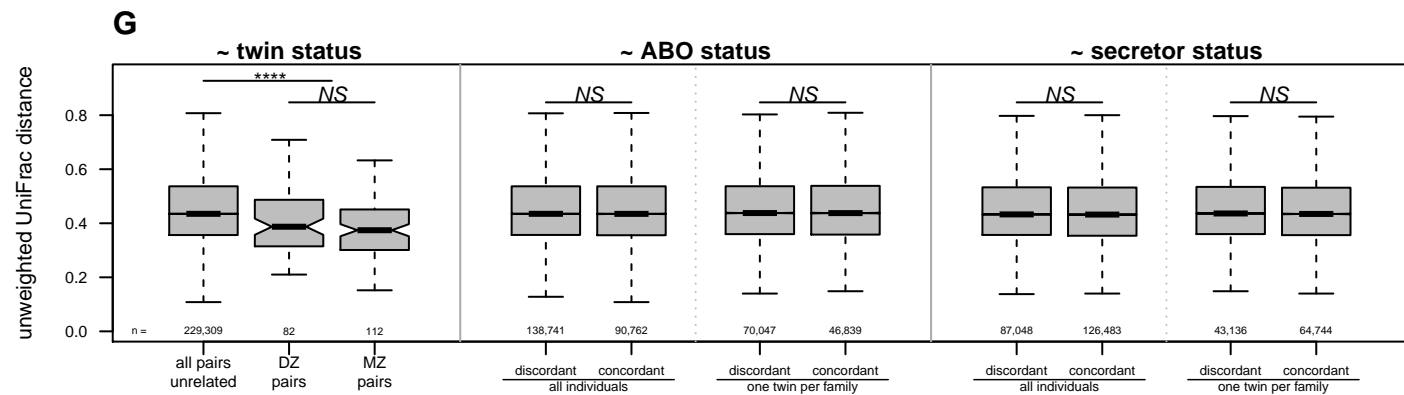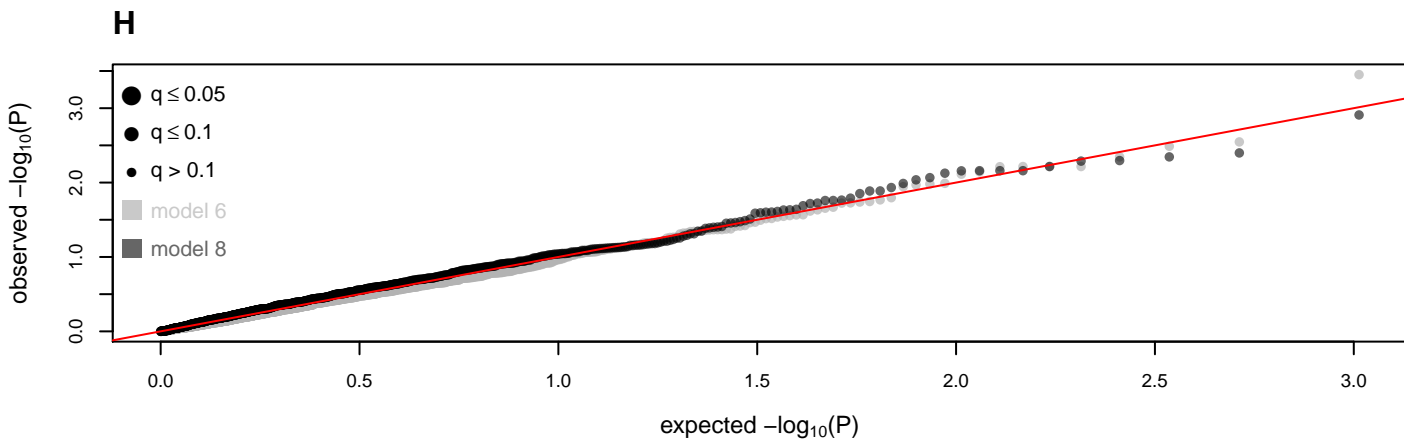

Supplement: Additional file 17: Figure S9. — Analyses using only individuals with BMI < 25 recapitulate results. A-C) Neither ABO or secretor status associated with broad compositional differences of the gut microbiota in the TwinsUK. None of the top 100 principal coordinates (PCs) from principal coordinate analysis of unweighted UniFrac distance are significantly associated with either ABO or secretor status. The first two PCs are shown, colored by ABO status (A) and secretor status (B). (C) Discriminant analysis of PCA (DAPC) is largely unsuccessful at predicting ABO or secretor status from microbiome data. The mean accuracy from 5-fold cross validation is plotted for ABO status, secretor status, and ABO status only in secreting individuals (yellow). Significance was determined by comparing the accuracy of each test to the accuracies of permuted data, which took into account twin relationships (gray). D-F) Microbiome diversity does not significantly differ by ABO, but does by secretor status. Within sample diversity (Faith’s phylogenic diversity) is significantly different between secretors versus non-secretors (D, P < 0.05), but not across the ABO groups in all individuals (E, P > 0.05), or across ABO groups in only secreting individuals (C, P > 0.05). (F) Microbiomes are more similar for siblings versus pairs of unrelated individuals, as measured by unweighted UniFrac distance. However, microbiomes of pairs of individuals concordant for either ABO or secretor status are not more similar than for pairs of individuals who are discordant. This holds true when all individuals in the dataset are considered (“all individuals”) or when only one individual from each twin pair is examined (“one twin per family”). The total number of pairs of individuals within each boxplot is indicated with “n = “. H) None of the common taxa are associated with ABO or secretor status. QQ-plot displaying the expected –log10(P-value) compared to the –log10(P-value) for all taxa tested in linear mixed models 6 (light gray points) and [file 12864_2016_3290_MOESM17_ESM.pdf]
